# Suppression of local invasion of ameloblastoma by inhibition of matrix metalloproteinase-2 in vitro

**DOI:** 10.1186/1471-2407-8-182

**Published:** 2008-06-30

**Authors:** Anxun Wang, Bin Zhang, Hongzhang Huang, Leitao Zhang, Donglin Zeng, Qian Tao, Jianguang Wang, Chaobin Pan

**Affiliations:** 1 Department of Oral and Maxillofacial Surgery, The First Affiliated Hospital, Sun Yat-sen University, Guangzhou, Guangdong, 510080, PR China; 2 Department of Oral and Maxillofacial Surgery, The Second Affiliated Hospital, Sun Yat-sen University, 107 Yan-jiang Road West, Guangzhou, Guangdong, 510120, PR China; 3 Department of Oral and Maxillofacial Surgery, Guanghua College of Stomatology, Sun Yat-sen University, Guangzhou, Guangdong, 510055, PR China

## Abstract

**Background:**

Ameloblastomas are odontogenic neoplasms characterized by local invasiveness. This study was conducted to address the role of matrix metalloproteinase-2 (MMP-2) in the invasiveness of ameloblastomas.

**Methods:**

Plasmids containing either MMP-2 siRNA or tissue inhibitor of metalloproteinase-2 (TIMP-2) cDNA were created and subsequently transfected into primary ameloblastoma cells. Zymography, RT-PCR, and Western blots were used to assess MMP-2 activity and expression of MMP-2 and TIMP-2, as well as protein levels.

**Results:**

Primary cultures of ameloblastoma cells expressed cytokeratin (CK) 14 and 16, and MMP-2, but only weakly expressed CK18 and vimentin. MMP-2 mRNA and protein levels were significantly inhibited by RNA interference (*P *< 0.05). Both MMP-2 siRNA and TIMP-2 overexpression inhibited MMP-2 activity and the *in vitro *invasiveness of ameloblastoma.

**Conclusion:**

These results indicate that inhibition of MMP-2 activity suppresses the local invasiveness of ameloblastoma cells. This mechanism may serve as a novel therapeutic target in ameloblastomas pursuant to additional research.

## Background

Ameloblastomas are the most frequently encountered tumors arising from odontogenic epithelium [1,2]. Although characterized as a benign neoplasm, ameloblastomas are locally invasive and frequently recrudescent tumors of the jaw [2]. Numerous studies have identified both genetic and molecular alterations in these odontogenic tumors of the epithelium [3,4], but the mechanisms underlying the local invasiveness of this neoplasm have yet to be clarified.

Matrix metalloproteinases (MMPs) are a family of zinc- and calcium-dependent proteolytic enzymes [4]. These enzymes play central roles in the regulation of the extracellular matrix during embryonic development and tissue remodeling. MMPs also participate in tumor invasion and metastasis [5,6]. The major function of tissue inhibitors of matrix metalloproteinases (TIMPs) is to inhibit the active forms of MMPs in a 1:1 stoichiometric ratio via non-covalent mechanisms [7,8]. Aberrant MMP activity in tumor cells and the surrounding stromal tissues has been implicated in tumor invasion and metastasis [9,10]. Previous studies have shown that ameloblastomas have an elevated expression of MMP-2, MMP-9, and vascular endothelial growth factor (VEGF), and are void of or have an abnormal expression of E-cadherin and TIMP-2 [11-14].

Therapeutic interventions that inhibit MMP activity appear to be promising based on a number of *in vitro *and *in vivo *tumor invasiveness studies [15-17]. To determine whether inhibition of MMP-2 activity is capable of suppressing the invasiveness of ameloblastomas, plasmids were constructed and subsequently transfected into ameloblastoma cells to cause the overexpression of, or to knockdown, MMP-2. This study was designed to test the hypothesis that MMP-2 activity is involved in the invasiveness of ameloblastomas and that inhibition of MMP-2 is a useful approach for treating ameloblastomas. The data collected in this study indicate that siRNA targeting of MMP-2 mRNA or TIMP-2 overxpression inhibits the activity of MMP-2 in ameloblastoma cells, which results in reduced ameloblastoma cell invasiveness *in vitro*, indicating that inhibition of MMP-2 activity may serve as a novel therapeutic target in the management of ameloblastomas.

## Methods

### Primary cell cultures and identification

The samples used in this study were obtained after obtaining informed consent of each patient and with the approval of the Sun Yet-sen University Ethics Committee. Briefly, ameloblastoma tissues were minced and incubated overnight in Dulbecco's modified Eagle medium (DMEM, Invitrogen, CA, USA) containing 1 mg/mL collagenase I (Invitrogen) at 37°C. Collagenase-digested tissues were plated onto 35 mm dishes coated with collagen I (Invitrogen) in DMEM containing 10% fetal calf serum, 200 μg/ml streptomycin, and 200 IU/ml penicillin, and incubated at 37°C with 5% CO_2_. When the cells were confluent, they were divided again and used for the ensuing experiments.

Immunocytochemistry was used to confirm the epithelial origin of the ameloblastoma cells using the SP method as described by the manufacturer (Maixin, Fuzhou, China). The primary antibodies were anti-cytokeratin 14, 16, and 18 and anti-vimentin (Maixin). Immunofluorescence was used to detect the expression of MMP-2, as described by the product's manufacturer.

### Plasmid construction and transient transfection

To generate the plasmid vector, pRNA-MMP-2, pRNA-U6.1/neovector (Genscript, NJ, USA) containing a cGFP sequence was used. MMP-2 shRNA contains a complement of a 21-nucleotide sequence (tgtgctgaaggacacactaaa, GenBank NM-004530), which was separated by a 7-nucleotide non-complementary spacer (CCACACC). A control vector (pRNA-neg) was constructed in the same way using a 21-nucleotide sequence (gattcaggtgtagaacgagca). These sequences were confirmed using nucleotide BLAST to ensure that there was no homology with any other known human gene. These annealed sequences were inserted into the pRNA-U6.1/neo backbone after digestion with BamH1 and HindIII. After amplification, all vector constructs were verified by sequencing.

The pcDNA3 vector (Genscript) containing an enhanced green fluorescence protein (EGFP) sequence was employed to generate the plasmid vector, pcDNA-TIMP-2. The cDNA encoding TIMP-2 was obtained via RT-PCR. The primer sequence for TIMP-2 (723 bp, GenBank NM003522) containing EcoRI and XhoI was as follows: forward 5'cgatgaattcatgggcgccgcggcccgc3'; reverse 5'cgatctcgagttattatgggtcctcgatgagaaac3'. TIMP-2 cDNA was subcloned into the clone site of the pcDNA3. Following amplification, vector constructs were verified by sequencing.

The constructed plasmids were transiently transfected into the cultured ameloblastoma cells using Lipofectamine Plus reagent (Invitrogen), according to the manufacturer's instructions. Transfected cells were subsequently used in the following experiment.

### Detection of MMP-2/TIMP-2 activity

MMP-2/TIMP-2 activity in the culture medium of the ameloblastoma cells was detected by zymography according to the method reported by Kleiner *et al. *[18]. Briefly, 10 μl of the medium from serum-free ameloblastoma cell cultures was mixed with the same volume of sample buffer and applied to a 10% (wt/vol) polyacrylamide gel containing 1 mg/ml gelatin. Gels were incubated in 2.5% Triton X-100 for 45 min after electrophoresis, then incubated at 37°C overnight in a digestion buffer. Gels were stained and destained. The bands were analyzed by an auto-imaging analysis system (Kontron IBAS2.0, Germany). All densitometry measurements were made between samples in the same gel to ensure comparability.

For detection of TIMP-2 activity, all procedures were similar to the detection of MMP-2 activity (described above), except that the gel contained 1% (wt/vol) MMP-2 (Sigma, St. Louis, MO, USA).

### RNA preparation and RT-PCR

Total RNA was extracted using an RNeasy mini kit (Qiagen), according to the manufacturer's instructions. RT-PCR was performed using one-step RT-PCR assays (Qiagen). Specific primers for detecting mRNA transcripts of the MMP-2 or TIMP-2 gene were as follows: MMP-2 (NM004530), 5'-AGCCACCCCTAAAGAGATCC-3' and 3'GTTCTAAGGCAGCCAGCAGT-5'; TIMP-2 (NM003255), 5'-ATTTGACCCAGAGTGGAACG-3' and 3'-TCCTTCGGCGAGTTTATGGA-5'; and GAPDH (NM008084), 5' GGTCGGAGTCAACGGATTTGGTCG-3' and 3'-CCTCCGACGCCTGCTTCACCAC-5'.

Transcript levels were normalized according to GAPDH transcripts and the products were resolved by agarose electrophoresis. The intensity was quantified by image-analysis computer software (NIH Image).

### Western blots

Western blotting was performed to detect MMP-2 and TIMP-2 proteins (Santa Cruz Biotechnology, Santa Cruz, CA, USA). Cells were harvested by trypsinization and lysed with a radio-immune precipitation assay (RIPA) lysis buffer (Santa Cruz Biotechnology). Total protein concentrations were measured by the Bradford method (Bio-Rad). Aliquots (30–50 μg) of cellular proteins were resolved by SDS-PAGE (10%), then electrotransferred onto PVDF membranes and immunoprobed. The protein-antibody complexes were detected by chemiluminescence (CSPD; Tropix, Bedford, MA, USA), according to the manufacturer's protocol (Applied Biosystems, MA, USA). The GAPDH gene was used as an internal control and the band intensity was quantified.

### In vitro cell invasion

The invasive ability of ameloblastoma cells was assayed in transwell cell chambers (Costa, Cambrige, MA, USA), according to the method reported by Kido *et al. *[19]. Briefly, polycarbonate filters with an 8.0 μm pore size were precoated with fibronectin on the lower surface. Matrigel was applied to the upper surface of the filters (5 μg/filter). Ameloblastoma cell suspensions (100 μl with 2 × 10^6 ^cells/ml) that had or had not been transfected were added to the upper compartment and incubated for 72 h at 37°C at 5% CO_2_. The filters were fixed with methanol and stained with Giemsa stain. The cells invading the lower surface through the Matrigel were manually counted under a microscope. The rate of invasion was calculated by the following equation: (number of cells invading the lower surface in the control group – number of cells invading the lower surface in the treated group)/number of cells invading the lower surface in the control group × 100%.

### Statistical analysis

All experiments were performed in triplicate. Data are expressed as the mean ± standard deviation (SD). Analysis of variance (ANOVA) was used to compare differences between treatment and control cells. A *p *< 0.05 was considered significant.

## Results

### Cells in primary culture

**Figure 1:**
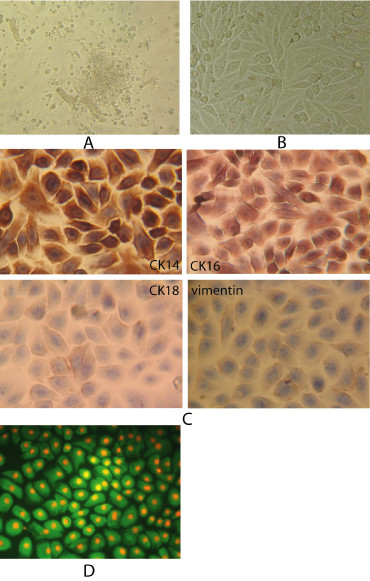
**Primary cultures of ameloblastoma cells**. (A) Primary culture ameloblastoma cells formed a colony or migrated out from ameloblastoma tissue under phase-contrast microscopy (× 100). (B) Sub-cultured ameloblastoma cells had vigorous growth in 2–4 passages. Cells were large and flattened or spindle-shaped under phase-contrast microscopy (× 200). (C) Immunocytochemistry confirmed the epithelial origin of the ameloblastoma cells based on cytokeratin 14 and 16 expression; cytokeratin 18 and vimentin were only weakly expressed (× 300). (D) Immunofluorescence for MMP-2 in ameloblastoma cells. Cells were examined and photographed under fluorescent microscopy. The green fluorescence shows the localization of MMP-2 and nuclei were counterstained with propidium iodine (PI) which fluoresced red (× 300).

After ameloblastoma tissues were cultured for 18–36 hours, ameloblastoma cells either formed a colony or migrated out from the ameloblastoma tissue and underwent membraniform growth in which cells were arranged like a slabstone (Figure 1A). Secondary cell cultures showed large, flattened, or spindle-shaped morphology (Figure 1B). Immunocytochemistry revealed that the cultured ameloblastoma cells expressed normal levels of cytokeratins 14 and 16 and only weakly expressed cytokeratin 18 and vimentin (Figure 1C), indicating that these cultures were primarily comprised of ameloblastoma cells of epithelial origin.

To investigate whether MMP-2 was expressed in the primary ameloblastoma cell cultures, immunofluorescence was used. Ameloblastoma cells that were immunopositive for MMP-2 showed green fluorescence in the cytoplasm and red fluorescence in the cell nucleus under a fluorescent microscope (Figure 1D).

### MMP-2 siRNA transfection decreased MMP-2 expression and activity

**Figure 2:**
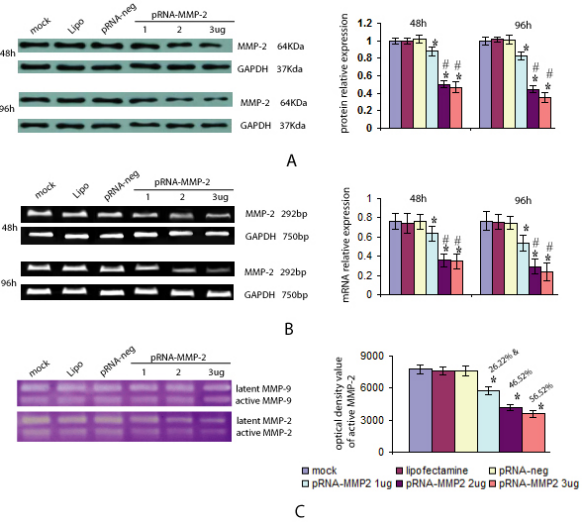
**Effect of MMP-2 siRNA on ameloblastoma cells**. Ameloblastoma cells were transfected with mock, lipofectamine, pRNA-neg, pRNA-MMP-2 (1 μg), pRNA-MMP-2 (2 μg), and pRNA-MMP-2 (3 μg) for the indicated times. GAPDH was used as an internal control to normalize the amounts of protein or mRNA. The relative protein or mRNA level of MMP-2 was depicted as the ratio of the density of MMP-2 to GAPDH for the same time point. The results shown are representative of triplicate experiments. *Compared with the mock, lipifectamine or vector controls, respectively, *p *< 0.05; #Compared with the 1 μg plasmid transfection group, *p *< 0.05. (A) Western blots were used to analyze MMP-2 protein levels in MMP-2 siRNA-transfected ameloblastoma cells. MMP-2 siRNA transfection decreased MMP-2 protein expression levels in a dose- and time-dependent manner compared with mock and scrambled vector controls. (B) RT-PCR was employed to detect the transcription of MMP-2 after MMP-2 siRNA transfection. Transcript levels of MMP-2 in MMP-2 siRNA-transfected cells were significantly reduced in a dose- and time-dependent manner compared with cells transfected with mock and lipofectamine and pRNA-neg. (C) Zymographic analysis for MMP-2 activity in the conditioned medium of ameloblastoma cells transfected with pRNA-MMP-2 for 96 h. Both latent and active form of MMP-2 and MMP-9 were detected in the gelatin zymogram. MMP-2 siRNA transfection inhibited MMP-2 activity in a dose-dependent manner in ameloblastoma cells.

To investigate whether MMP-2 siRNA transfection decreases MMP-2 expression and activity, ameloblastoma cells were transfected with various doses of pRNA-MMP-2 (1–3 μg) and the protein level of MMP-2 was analyzed by Western blots. MMP-2 siRNA transfection decreased MMP-2 protein expression in both a dose- and time-dependent manner compared with mock and scrambled vector controls (Figure 2A). There were no differences between the three control groups (*p *> 0.05), but a significantly lower protein level of MMP-2 after transfection with various doses of pRNA-MMP-2 (*p *< 0.05) were noted compared with the three control groups. This MMP-2 inhibition was more pronounced in the cells transfected with 2 or 3 μg of plasmid than in the 1 μg plasmid transfection group (*p *< 0.05). No difference between the 2 and 3 μg plasmid transfection groups was noted (*p *> 0.05).

To determine whether the decreased production of MMP-2 was due to a decrease in gene transcription, MMP-2 transcripts were assessed via RT-PCR. As shown in Figure 2B, the transcript levels of MMP-2 in MMP-2 transfected cells were significantly lower than MMP-2 RNA levels in the mock, lipidosome, and pRNA-negative transfected cells(*p *< 0.05).

To investigate whether MMP-2 activity was associated with MMP-2 knockdown, a zymogram assay was used to detect enzyme activity in the culture medium following transfection with various doses of pRNA-MMP-2 for 96 h (Figure 2C). Both latent and active forms of MMP-2 and MMP-9 were detected in the gelatin zymogram. The MMP-9 was not different after MMP-2 knockdown compared to pre-knockdown values. MMP-2 siRNA transfection inhibited MMP-2 activity in a dose-dependent manner whereas no difference in MMP-2 activity was identified in the three control groups (*p *> 0.05). After transfection of ameloblastoma cells with various doses of pRNA-MMP-2, the activity of MMP-2 was significantly decreased compared with the three control groups (*p *< 0.05).

### Overexpression of TIMP-2 inhibited MMP-2 activity

**Figure 3:**
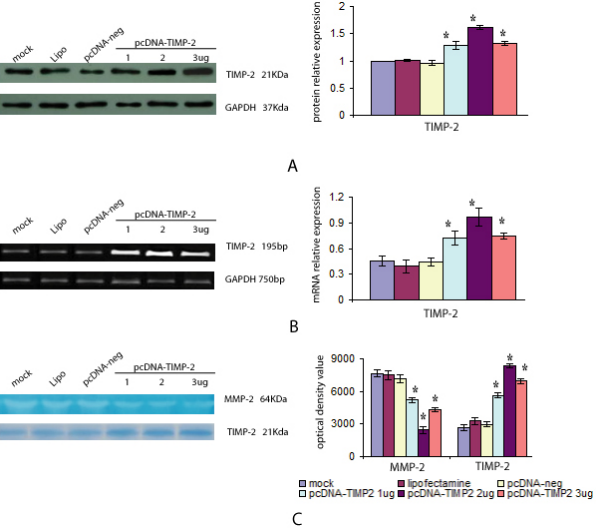
**Overexpression of TIMP-2 inhibited the MMP-2 activity of ameloblastoma cells**. Ameloblastoma cells were transfected with mock, lipofectamine, pcDNA-neg, pcDNA-TIMP-2 (1 μg), pcDNA-TIMP-2 (2 μg), or pcDNA-TIMP-2 (3 μg) for 72 h. GAPDH was included as an internal control to normalize the amounts of protein or mRNA. The relative protein or mRNA level of TIMP-2 was depicted as the ratio of the density of TIMP-2 to GAPDH for the same time point. The results are a representative experiment among three. *Compared with the mock, lipifectamine or vector controls, respectively, *p *< 0.05. (A) Western blotting (WB) was used to detect TIMP-2. TIMP-2 protein levels increased in ameloblastoma cells after transfection with pcDNA-TIMP-2. (B) RT-PCR was used to detect the transcription of TIMP-2 after pcDNA-TIMP-2 transfection. Transcript levels of TIMP-2 in TIMP-2-transfected cells were significantly higher compared with cells transfected with mock and lipofectamine and pcDNA-neg. (C) Zymographic analysis for MMP-2 and TIMP-2 activity in the conditioned medium of ameloblastoma cells transfected with various doses of pcDNA-TIMP-2. The zymography assay revealed that pcDNA-TIMP-2 transfection decreased MMP-2 activity. The inhibition rates were 31.88, 67.87, and 43.57% for 1, 2, and 3 μg, respectively. Reverse gelatin zymography showed that pcDNA-TIMP-2 transfection increased TIMP-2 activity.

To determine the effect of TIMP-2 overexpression on MMP-2 activity, ameloblastoma cells were transfected with various doses of pcDNA-TIMP-2 (1–3 μg). Western blots and RT-PCR showed that the levels of both TIMP-2 mRNA and TIMP-2 protein in the TIMP-2 transfected cells were significantly higher than that of cells transfected with mock, lipofectamine, and pcDNA-neg (*p *< 0.05; Figures 3A and 3B). Further, zymography and reverse gelatin zymography assays revealed that pcDNA-TIMP-2 transfection decreased MMP-2 activity and increased TIMP-2 activity in ameloblastoma cells (Figure 3C). While no difference between the three control groups were noted (*p *> 0.05), significantly lower MMP-2 activity and significantly higher TIMP-2 activity were noted following transfection with various doses of pcDNA-TIMP-2 (*p *< 0.05).

### Effect of MMP-2 siRNA or TIMP-2 overexpression on the invasiveness of ameloblastoma cells

The effect of MMP-2 knockdown and TIMP-2 overexpression on ameloblastoma invasion was assessed using Matrigel-coated transwells and the results are summarized in Table 1. MMP-2 siRNA and pcDNA-TIMP-2 each inhibited the invasion of ameloblastoma cells through the Matrigel compared to the mock, lipifectamine, and vector-transfected control cells. While there was no difference between the invasion of cells in the three control groups (*p *> 0.05), significantly fewer cells invaded through the Matrigel after transfection with various doses of pcDNA-TIMP-2 or pRNA-MMP-2 (*p *< 0.05) compared with the three control groups. Further, a significant difference in inhibition of invasion was noted between the cells transfected with 1, 2, or 3 μg of pRNA-MMP-2 or pcDNA-TIMP-2, but no significant differences existed between cells transfected with 2 or 3 μg of pRNA-MMP-2 or pcDNA-TIMP-2. Specifically, the invasiveness of ameloblastoma cells after transfection with 1, 2 or 3 μg of pRNA-MMP-2 was decreased by 15.3, 55.5, and 61.3%, respectively, and 24.2, 53.3, and 50.5% in cells transfected with pcDNA-TIMP-2, respectively.

**Table 1:** Analysis of the effect of MMP-2 siRNA or TIMP-2 overexpression on ameloblastoma cell invasion.

Group	Invaded cells (Cells/Well)	Inhibition rate of invasion
MMP-2siRNA		
Mock	137 ± 21	0
Lipofectamine	129 ± 18	5.8
pRNA-neg	131 ± 28	4.4
pRNA-MMP-2(1 μg)	116 ± 24*	15.3
pRNA-MMP-2(2 μg)	61 ± 16*	55.5
pRNA-MMP-2(3 μg)	53 ± 20*	61.3
TIMP-2 overexpression		
Mock	128 ± 18	0
Lipofectamine	123 ± 21	3.9
pcDNA-neg	117 ± 16	8.6
pcDNA-TIMP-2(1 μg)	97 ± 25*	24.2
pcDNA-TIMP-2(2 μg)	59.8 ± 23*	53.3
pcDNA-TIMP-2(3 μg)	63.4 ± 19*	50.5

*Compared with the mock, lipifectamine or vector controls, respectively, *P *< 0.05.

## Discussion

The goal of this study was to determine whether inhibition of MMP-2 activity was capable of suppressing the local invasiveness of human ameloblastoma cells. This was accomplished using an MMP-2 gene knockdown approach or TIMP-2 overexpression and subsequently detecting the relationship between MMP-2 activity and the local invasiveness of ameloblastoma cells.

To date, three approaches for targeting MMP-2 activity have been utilized: 1) the *in vitro *and *in vivo *delivery of naturally occurring inhibitors of MMP-2 (i.e., TIMP); 2) the design and delivery of novel inhibitory molecules or modifications of naturally occurring inhibitors; and 3) targeting MMP-2 mRNA via various gene silencing strategies [20]. Previous studies performed by our group revealed that the MMP-2 inhibitor, Ro31-9790, inhibited adhesion and invasion of ameloblastoma cells in primary cell cultures [21]. While Ro31-9790 did not alter the expression of either MMP-2 or TIMP-2, Ro31-9790 did inhibit the activity of MMP-2. This led to the supposition that the suppression of the local invasiveness of ameloblastoma cells may be related to inhibition of MMP-2 activity.

Utilization of siRNAs is one of the most effective gene silencing methods and is a promising new approach in the analysis of gene function and gene therapy [22-25]. Numerous studies have used siRNAs to analyze the function of MMP-2 [26-28], but to date no studies have utilized this technique with ameloblastomas. Therefore, we investigated whether siRNAs targeted at MMP-2 are capable of inhibiting the activity of MMP-2 in ameloblastoma cells. siRNAs against MMP-2 significantly inhibited MMP-2 mRNA expression and MMP-2 protein levels in primary ameloblastoma cell cultures. Furthermore, MMP-2siRNA transfection decreased the activity of MMP-2.

The conversion of MMP proenzymes to the activated forms is controlled by the stoichiometric binding of TIMPs which are synthesized by cells, such as fibroblasts, endothelial cells, and tumor cells [29]. The ability of TIMPs to inhibit the activity of MMPs is known to significantly suppress tumor invasion and metastasis [5,30,31]. To determine whether TIMP-2 inhibited the activity of MMP-2 in ameloblastoma cells, plasmids were constructed to overexpress TIMP-2. TIMP-2 overexpression inhibited MMP-2 activity in ameloblastoma cells. This outcome suggested that TIMP-2 might suppress invasiveness in ameloblastomas in humans.

The underlying molecular mechanisms resulting in local invasion by ameloblastomas are closely related to the proteolytic degradation of the basement membrane. Among the proteases thought to be involved in ameloblastoma invasion, attention has focused on MMP-2 [11,12,14,21]. To investigate whether inhibition of MMP-2 activity will suppress the invasiveness of ameloblastoma cells, we knocked down MMP-2 by RNA interference or overexpressed TIMP-2. Both MMP-2 knockdown and TIMP-2 overexpression inhibited the activity of MMP-2. The invasion assay showed that the ability of ameloblastoma cells to invade the lower surface of the filter through the Matrigel was significantly inhibited in both MMP-2 knockdown or TIMP-2 overexpression cells compared to the control cultures.

## Conclusion

In summary, these data indicate that siRNA targeting of MMP-2 mRNA or TIMP-2 overxpression using a plasmid-based system effectively inhibited the activity of MMP-2 in ameloblastoma cells, which subsequently resulted in reduced ameloblastoma cell invasiveness *in vitro*. This study provided evidence that inhibition of MMP-2 activity may serve as a novel therapeutic target in the clinical management of ameloblastoma. Further research is warranted.

## List of abbreviations used

MMP: matrix metalloproteinase; TIMP: tissue inhibitor of metalloproteinase; CK: cytokeratin; DMEM: Dulbecco's modified Eagle medium; ANOVA: one-way analysis of variance; VEGF: vascular endothelial growth factor; GFP: green fluorescence protein; GAPDH, glyceraldehyde-3-phosphate dehydrogenase; RIPA: radio-immune precipitation assay; shRNA: small hairpin RNA; siRNA: small interference RNA; mRNA: message RNA; RT-PCR: reverse transcription polymerase chain reaction; SDS-PAGE: sodium dodecyl sulfate polyacrylamide gel electrophoresis.

## Competing interests

The authors declare that they have no competing interests.

## Authors' contributions

AW, BZ, LZ, DZ, QT, and HH were responsible for the experimental design and completion of all laboratory work represented in this manuscript. JW and CP participated in the design and coordination of the work involved. The manuscript was drafted by AW and BZ. All authors have read and approved the final manuscript

## Pre-publication history

The pre-publication history for this paper can be accessed here:


